# Comparison of Stroke Recurrence, Cardiovascular Events, and Death Among Patients With Pregnancy-Associated vs Non–Pregnancy-Associated Stroke

**DOI:** 10.1001/jamanetworkopen.2023.15235

**Published:** 2023-06-07

**Authors:** Yannick Béjot, Valérie Olié, Gregory Lailler, Clémence Grave, Nolwenn Regnault, Gauthier Duloquin, Jacques Blacher, Amélie Gabet

**Affiliations:** 1 Dijon Stroke Registry, University Hospital and Medical School of Dijon, University of Burgundy, Burgundy, France; 2 Santé Publique France, Saint-Maurice, France; 3 Diagnosis and Therapeutic Center, Hotel Dieu, Assistance Publique–Hôpitaux de Paris, University Paris Descartes, Paris, France

## Abstract

**Importance:**

Women with pregnancy-associated stroke might have different risks of stroke recurrence, including during subsequent pregnancies, and other cardiovascular events due to pregnancy-specific stroke risk factors, such as gestational hypertension, preeclampsia, or gestational diabetes.

**Objective:**

To estimate the rate of stroke recurrence, cardiovascular hospitalization, and death in women with pregnancy-associated stroke compared with women with non–pregnancy-associated stroke.

**Design, Setting, and Participants:**

This cohort study included all women aged 15 to 49 years in France who were affiliated with the general scheme of French health care insurance (94% of women) and had a first hospitalization for stroke between January 1, 2010, and December 31, 2018. Women were followed up until December 31, 2020, with the registration of stroke recurrence, hospitalization for cardiovascular conditions, and death. Data were from the French health care database Système National des Données de Santé. Statistical analyses were conducted between December 2021 and September 2022.

**Exposure:**

Pregnancy status at the time of stroke.

**Main Outcomes and Measures:**

Poisson regressions were used to estimate incidence rates of these events with 95% CIs. We used Cox proportional hazards regression models to estimate the hazard ratios (HRs) of each event during the follow-up for women with a pregnancy-associated stroke vs women with a non–pregnancy-associated stroke.

**Results:**

Among women aged between 15 and 49 years between 2010 and 2018 and living in France, 1204 had a pregnancy-associated stroke at a mean (SD) age of 31.5 (5.8) years, and 31 697 had a non–pregnancy-associated stroke at a mean age of 39.6 (8.2) years. Among the 1204 women with a pregnancy-associated stroke, the incidence rate was 11.4 (95% CI, 9.0-14.3) per 1000 person-years, with 2 recurrent events during a subsequent pregnancy. Compared with women with non–pregnancy-associated stroke, women with pregnancy-associated stroke had lower risks of ischemic stroke (adjusted HR, 0.53; 95% CI, 0.36-0.77), cardiovascular events (adjusted HR, 0.58; 95% CI, 0.49-0.69), and death (adjusted HR, 0.42; 95% CI, 0.22-0.79). In contrast, the risk of recurrent intracerebral hemorrhage and cerebral venous thrombosis did not differ significantly, whereas the risks of venous thromboembolism (HR, 2.02; 95% CI, 1.14-3.58) and acute coronary syndrome with ST-segment elevation (HR, 3.93; 95% CI, 1.10-14.0) were increased.

**Conclusions and Relevance:**

In this cohort study, although the risks of ischemic stroke, overall cardiovascular events, and mortality were lower after a pregnancy-associated stroke than after a non–pregnancy-associated stroke, the risks of venous thromboembolism and acute coronary syndrome with ST-segment elevation were higher. Recurrent stroke during a subsequent pregnancy remained rare.

## Key Points

**Question:**

Do stroke recurrence, cardiovascular events, and death risk vary after pregnancy-associated stroke compared with non–pregnancy-associated stroke in women of the same age?

**Findings:**

In this cohort study, compared with women with non–pregnancy-associated stroke, women with pregnancy-associated stroke had lower risks of ischemic stroke, overall cardiovascular events, and death and similar risks of intracerebral hemorrhage and cerebral venous thrombosis, whereas the risks of venous thromboembolism and acute coronary syndrome with ST-segment elevation were greater.

**Meaning:**

These results suggest that transitory pregnancy-related risk factors and persistent risk factors may be associated with, respectively, a lower risk of stroke recurrence and a higher risk of venous thromboembolism and acute coronary syndrome with ST-segment elevation among individuals with pregnancy-associated stroke vs non–pregnancy-associated stroke.

## Teaser

This cohort study investigates whether the incidence rates of recurrent stroke, cardiovascular events, and mortality are different among women with pregnancy-associated stroke compared with women with non–pregnancy associated stroke.

## Introduction

Stroke during pregnancy and post partum is a rare but potentially devastating disease. A meta-analysis estimated a rate of pregnancy-associated stroke of 30 per 100 000 pregnancies.^1^ In France, a recent study estimated that 24 per 100 000 person-years experienced a first-ever stroke during pregnancy, in peripartum, or in the first 6 weeks post partum.^2^ Other studies have shown that the incidence of pregnancy-associated ischemic stroke (IS) and hemorrhagic stroke (HS) is increasing over time.^2,3^ The distribution of stroke causes varies greatly between pregnant and nonpregnant young women, with a higher proportion of intracerebral hemorrhage (ICH) and cerebral venous thrombosis (CVT) in pregnant women.^2^ Specific risk factors during pregnancy may contribute to these differences. In addition, the risk of recurrence might differ between women with pregnancy-associated and non–pregnancy-associated stroke. However, few data are available in the literature, and previous studies were limited by small samples of patients.^4,5,6,7^

To address this issue, this study aimed to estimate the incidence rates of recurrent stroke, cardiovascular events, and mortality among women with pregnancy-associated stroke and among women with non–pregnancy-associated stroke. The risk of each event was compared regarding pregnancy-associated status of the initial stroke. A secondary objective was to analyze the recurrence of stroke during a subsequent pregnancy.

## Methods

### Data Sources

This study was based on the French health care database Système National des Données de Santé (SNDS).^8^ This database collected all reimbursed health care expenditures, including hospitalizations, at an individual level for people living in France (ie, approximately 66 million of inhabitants). According to the French governmental regulations and the National Ethics Committee, no patient consent was required. The databases used in the study contained pseudonymized patient information. Furthermore, full access to the SNDS, which includes the French National Hospital databases, is granted to the National Agency for Public Health (Santé Publique France) by decree. Finally, our study followed the Strengthening the Reporting of Observational studies in Epidemiology (STROBE) reporting guidelines.

### Study Patients

A flowchart of the study population’s selection is provided in Figure. All women aged 15 to 49 years who were hospitalized for acute stroke between January 1, 2010, and December 31, 2018, in France were included in the study (eMethods in Supplement 1). Women with a history of cerebrovascular events between 2006 and 2009 were excluded in an effort to include only women with incident stroke in the study. Women affiliated with a health insurance scheme other than the general one were excluded (6.3%) because of incomplete data.

**Figure.**
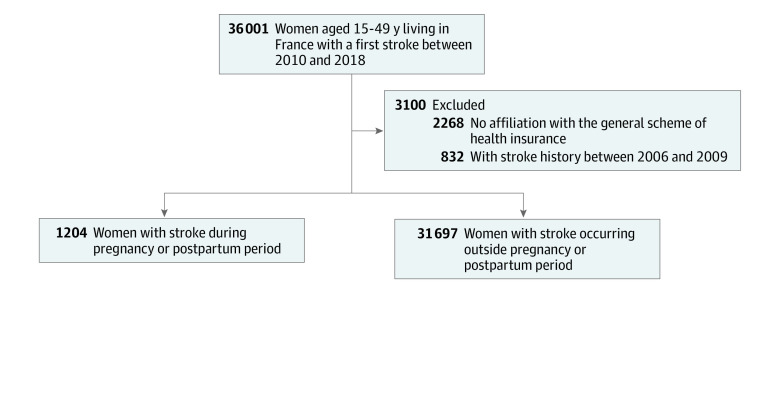
Flowchart of Patient Enrollment Stroke cases that occurred during pregnancy (≥22 weeks of amenorrhea at delivery) or post partum (≤6 weeks after delivery) were identified from the CONCEPTION (Cohort of Cardiovascular Diseases in Pregnancy) study cohort (n = 6 298 967), which includes all French women aged 15 to 49 years who gave birth in France between January 1, 2010, and December 31, 2018.

Patients were divided into 2 groups: women with a stroke occurring during pregnancy or post partum and women with a non–pregnancy-associated stroke. Selected stroke cases that arose in pregnant women (≥22 weeks of amenorrhea at delivery) or post partum (≤6 weeks after delivery) were identified from the CONCEPTION (Cohort of Cardiovascular Diseases in Pregnancy) study, which is a prospective cohort from the same SNDS database that includes all French women who gave birth in France between January 1, 2010, and December 31, 2018.^9^ For the secondary objective, women who had a subsequent pregnancy recorded in the CONCEPTION cohort were identified. Among them, pregnancy-associated strokes were searched for.

### Follow-up and Outcomes

All women were followed up from hospital discharge until December 31, 2020. Hospitalizations for stroke or cardiovascular events (see eMethods in Supplement 1 for detailed codes) and deaths were recorded during the follow-up. A subsequent hospitalization for stroke in the first 30 days was considered as an early recurrent stroke. After 30 days, hospitalizations for stroke were considered as recurrent strokes. The women’s recorded characteristics are given in the eMethods in Supplement 1.

### Statistical Analysis

Analyses were conducted between December 2021 and September 2022. The incidence rates of recurrent stroke, cardiovascular events, and death were estimated using Poisson regressions. Incidence rates were reported with 95% CIs. Kaplan-Meier curves were computed. Cox proportional hazards regression models with age as the time scale^10^ were used to estimate the hazard ratios (HRs) of each event for pregnancy-associated stroke compared with non–pregnancy-associated stroke (eMethods in Supplement 1). To assess the potential role of hypertensive disorders of pregnancy (HDP) in stroke recurrence, cardiovascular incidence, and death occurrence, 3 complementary analyses were performed and are presented in the eMethods in Supplement 1. We performed a matched cohort sensitivity analysis by selecting for each woman with pregnancy-associated stroke 5 women with non–pregnancy-associated stroke of the same age and Charlson Comorbidity Index score. Models 1 to 3 were computed with this matched population and HR of each event of interest between pregnancy-associated and non–pregnancy-associated stroke were given. Statistical analysis was performed with SAS software, version 7.11 (SAS Institute Inc).

## Results

Between 2010 and 2018, a total of 1204 pregnancy-associated strokes and 31 697 non–pregnancy-associated strokes were recorded in women aged 15 to 49 years. The mean (SD) age at time of stroke was 31.5 (5.8) years for pregnancy-associated stroke and 39.6 (8.2) years for non–pregnancy-associated stroke. The distribution of IS, HS, and CVT differed significantly between the 2 groups, with 493 ISs (41.0%), 486 HSs (40.4%), and 196 CVTs (16.3%) in women with pregnancy-associated stroke and 19 162 ISs (60.5%), 11 701 HSs (36.9%), and 545 CVTs (1.7%) in women with non–pregnancy-associated strokes (Table 1). eTable 1 in Supplement 1 presents the characteristics of included pregnancy-associated and non–pregnancy-associated strokes according to type of stroke.

**Table 1.** Characteristics of Women With Pregnancy-Associated Strokes, Non–Pregnancy-Associated Stroke, and Recurrent Stroke^a^

Characteristic	All women with a first stroke			Women with recurrent stroke		
	Pregnancy status at the first stroke, No. (%)		*P* value	Pregnancy status at the first stroke, No. (%)		*P* value
	Pregnant or post partum (n = 1204)	Nonpregnant (n = 31 697)		Pregnant or post partum (n = 76)	Nonpregnant (n = 2742)	
Age, y						
Mean (SD)	31.5 (5.8)	39.6 (8.2)	<.001	32.5 (6.2)	40.1 (7.9)	<.001
Median (IQR)	32 (28-36)	42 (35-46)		34 (28-37)	42 (36-46)	
Age groups, y						
15-29	455 (37.8)	4369 (13.8)	NA	23 (30.2)	341 (12.4)	NA
30-39	645 (53.6)	8066 (25.5)		45 (59.2)	674 (24.6)	
40-49	104 (8.6)	19 262 (60.7)		8 (10.6)	1727 (63.0)	
Tobacco smoking	118 (9.8)	5693 (18.0)	<.001	7 (9.2)	493 (18.0)	.05
Obesity	74 (6.2)	2568 (8.1)	.01	5 (6.6)	234 (8.5)	.55
Quintile of social deprivation of the town of living						
1 (Least deprived)	208 (18.8)	5067 (17.5)	.13	17 (24.6)	487 (19.8)	.54
2	238 (21.5)	5482 (18.9)		15 (21.7)	437 (17.8)	
3	214 (19.4)	5744 (19.8)		15 (21.7)	467 (19.0)	
4	207 (18.7)	5927 (20.4)		9 (13.0)	497 (20.2)	
5 (Most deprived)	238 (21.5)	6808 (23.4)		13 (18.8)	573 (23.3)	
Missing	99	2669		7	281	
Subtype of the first stroke						
Ischemic stroke, excluding CVT	493 (41.0)	19 162 (60.5)	<.001	32 (42.1)	1564 (57.0)	<.001
CVT	196 (16.3)	545 (1.7)	<.001	4 (5.3)	30 (1.1)	<.001
Hemorrhagic stroke (all)	486 (40.4)	11 701 (36.9)	.008	41 (54.0)	1156 (42.2)	<.001
ICH	260 (21.6)	4592 (14.5)	<.001	29 (38.2)	498 (18.2)	<.001
Subarachnoid hemorrhage	234 (19.4)	7188 (22.7)	.008	15 (19.7)	692 (25.2)	.27
In-hospital death	28 (2.3)	1940 (6.1)	<.001	NA	NA	NA
Stroke symptoms						
Plegia (hemiplegia and paraplegia)	323 (26.8)	10 185 (32.1)	<.001	35 (46.0)	1061 (38.7)	.19
Aphasia	220 (18.3)	6923 (21.8)	.003	22 (28.9)	671 (24.5)	.37
Epilepsy	110 (9.1)	1600 (5.1)	<.001	9 (11.8)	179 (6.5)	.07
Migraine	47 (3.9)	1394 (4.4)	.41	6 (7.9)	96 (3.5)	.04
Other migraine symptoms	56 (4.7)	585 (1.9)	<.001	4 (5.3)	60 (2.2)	.08
Headache	224 (18.6)	4392 (13.9)	<.001	14 (18.4)	397 (14.5)	.34
History of cardiovascular treatment in the year before the index stroke						
Antihypertensives	52 (4.4)	4923 (16.8)	<.001	4 (5.6)	565 (22.2)	<.001
Lipid-lowering medications	7 (0.6)	1474 (5.0)	<.001	0	209 (8.2)	.013
Antidiabetics	22 (1.9)	1089 (3.7)	<.001	1 (1.3)	168 (6.6)	.08
Oral anticoagulants	15 (1.3)	627 (2.1)	.04	1 (1.3)	82 (3.2)	.38
All anticoagulants	49 (4.2)	892 (3.0)	.03	1 (1.3)	99 (3.9)	.27
Antiplatelets	19 (1.6)	1148 (3.9)	<.001	2 (2.6)	161 (6.3)	.22
Antiarrhythmics	5 (0.4)	378 (1.3)	.009	1 (1.3)	35 (1.4)	.99
Heparin	44 (3.8)	291 (1.0)	<.001	1 (1.3)	25 (1.0)	.73
Comorbidities (5 y of history)						
Charlson Comorbidity Index score (No. of weighted comorbidities)						
0	859 (71.4)	19 489 (61.5)	<.001	39 (51.3)	1480 (54.0)	.87
1	10 (0.8)	841 (2.7)		1 (1.3)	99 (3.6)	
2	301 (25.0)	9489 (29.9)		33 (43.4)	962 (35.1)	
3	9 (0.8)	614 (1.9)		1 (1.3)	76 (2.8)	
≥4	25 (2.1)	1264 (4.0)		2 (2.6)	125 (4.6)	
Ischemic heart disease	12 (1.0)	1114 (3.5)	<.001	1 (1.3)	119 (4.3)	.20
Acute coronary syndrome	7 (0.6)	519 (1.6)	.004	1 (1.3)	67 (2.4)	.53
Atrial fibrillation	14 (1.2)	643 (2.0)	.03	0	74 (2.7)	.15
All heart rhythm disorders	43 (3.6)	1927 (6.1)	<.001	2 (2.6)	183 (6.7)	.16
Heart failure	18 (1.5)	869 (2.7)	.009	1 (1.3)	103 (3.8)	.27
Pulmonary embolism	22 (1.8)	564 (1.8)	.90	1 (1.3)	47 (1.7)	.79
All venous thromboembolism diseases	71 (5.9)	1281 (4.0)	.001	3 (3.9)	117 (4.3)	.89
All hospitalization for circulation diseases (*ICD-10* code I)	100 (8.3)	5338 (16.8)	<.001	5 (6.6)	601 (21.9)	.001
Pregnancy-related disorders						
Hypertensive disorders of pregnancy	278 (23.1)	NA	NA	12 (15.8)	NA	NA
Gestational hypertension	190 (15.8)	NA	NA	9 (11.8)	NA	NA
Preeclampsia or eclampsia	149 (12.4)	NA	NA	4 (5.3)	NA	NA
HELLP syndrome	40 (3.3)	NA	NA	2 (2.6)	NA	NA
Gestational diabetes	112 (9.3)	NA	NA	2 (2.6)	NA	NA

Abbreviations: CVT, cerebral venous thrombosis; HELLP, hemolysis, elevated liver enzymes, and low platelets; ICH, intracerebral hemorrhage; *ICD-10*, *International Statistical Classification of Diseases and Related Health Problems, Tenth Revision*; NA, not applicable.

^a^ Data are presented as number (percentage) of women unless otherwise indicated.

### Stroke Recurrence

After a mean (SD) follow-up of 5.5 (3.1) years (median [IQR], 5.6 [3.1-8.1] years), 76 of 1204 women with pregnancy-associated stroke (6.3%) experienced a recurrent stroke, corresponding to an incidence rate of 11.4 (95% CI, 9.0-14.3) per 1000 person-years (Table 2). Among the 31 697 women with non–pregnancy-associated stroke, 2742 (8.7%) had a recurrent stroke after a mean (SD) follow-up of 4.8 (3.4) years (median [IQR], 4.7 [2.1-7.7] years; incidence rate, 17.9 [95% CI, 17.2-18.6] per 1000 person-years). Women with a pregnancy-associated stroke were followed up during 2 (1037 of 1204 [86.1%]), 4 (783 of 1204 [65.0%]), 6 (563 of 1204 [46.8%]), 8 (328 of 1204 [27.2%]), and 10 (89 of 1204 [7.4%]) years. Women with non–pregnancy-associated stroke were followed up during 2 (24 097 of 31 697 [76.0%]), 4 (17 938 of 31 697 [56.6%]), 6 (12 442 of 31 697 [39.3%]), 8 (7074 of 31 697 [22.3%]), and 10 (2264 of 31 697 [7.1%]) years.

**Table 2.** Number and Rates per 1000 PYs of Recurrent Stroke, Cardiovascular Events, and Death During the Follow-up of Pregnancy- and Non–Pregnancy-Associated Stroke

Event	All women with a first stroke (32 901 with 1204 pregnancy-associated strokes)		Women with a first ischemic stroke (19 655 with 493 pregnancy-associated ischemic strokes)		Women with a first CVT (741 with 196 pregnancy-associated CVT)		Women with a first ICH (4852 with 260 pregnancy-associated ICH)	
	No. (%)	Incidence rate per 1000 PY (95% CI)	No. (%)	Incidence rate per 1000 PY (95% CI)	No. (%)	Incidence rate per 1000 PY (95% CI)	No. (%)	Incidence rate per 1000 PY (95% CI)
All strokes								
Not pregnant or post partum	2742 (8.7)	17.9 (17.2-18.6)	1564 (8.2)	15.4 (14.6-16.2)	30 (5.5)	9.5 (6.4-13.5)	498 (10.8)	26.2 (24.0-28.7)
Pregnant or post partum	76 (6.3)	11.4 (9.0-14.3)	32 (6.5)	11.4 (7.8-16.0)	4 (2.0)	3.5 (1.0-9.0)	29 (11.2)	21.9 (14.7-31.4)
Ischemic strokes								
Not pregnant or post partum	1655 (5.2)	10.8 (10.3-11.3)	1439 (7.5)	14.2 (13.4-14.9)	26 (4.8)	8.2 (5.4-12.0)	96 (2.1)	5.1 (4.1-6.2)
Pregnant or post partum	35 (2.9)	5.3 (3.7-7.3)	29 (5.9)	10.3 (6.9-14.8)	2 (1.0)	1.8 (0.2-6.3)	3 (1.2)	2.3 (0.5-6.6)
Hemorrhagic strokes								
Not pregnant or post partum	1168 (3.7)	7.6 (7.2-8.1)	164 (0.9)	1.6 (1.4-1.9)	6 (1.1)	1.9 (0.7-4.1)	424 (9.2)	22.3 (20.3-24.6)
Pregnant or post partum	42 (3.5)	6.3 (4.6-8.5)	3 (0.6)	1.1 (0.2-3.1)	2 (1.0)	1.8 (0.2-6.3)	27 (10.4)	20.4 (13.4-29.7)
CVT								
Not pregnant or post partum	26 (0.1)	0.2 (0.1-0.2)	7 (<0.0)	0.1 (0.0-0.1)	16 (2.9)	5.0 (2.9-8.2)	3 (0.1)	0.2 (0.0-0.5)
Pregnant or post partum	2 (0.2)	0.3 (0.0-1.1)	0	0.0 (0.0-1.3)	1 (0.5)	0.9 (0.0-4.9)	1 (0.4)	0.8 (0.0-4.2)
ICH								
Not pregnant or post partum	490 (1.5)	3.2 (2.9-3.5)	95 (0.5)	0.9 (0.8-1.1)	3 (0.6)	0.9 (0.2-2.8)	320 (7.0)	16.9 (15.1-18.8)
Pregnant or post partum	30 (2.5)	4.5 (3.0-6.4)	2 (0.4)	0.7 (0.1-2.6)	0	0.0 (0.0-3.2)	25 (9.6)	18.9 (12.2-27.9)
Subarachnoid hemorrhage								
Not pregnant or post partum	613 (1.9)	4.0 (3.7-4.3)	48 (0.3)	0.5 (0.3-0.6)	1 (0.2)	0.3 (0.0-1.8)	100 (2.2)	5.3 (4.3-6.4)
Pregnant or post partum	14 (1.2)	2.1 (1.2-3.5)	1 (0.2)	0.4 (0.0-2.0)	1 (0.5)	0.9 (0.0-4.9)	6 (2.3)	4.5 (1.7-9.9)
Venous thromboembolism								
Not pregnant or post partum	304 (1.0)	2.0 (1.8-2.2)	187 (1.0)	1.8 (1.6-2.1)	13 (2.4)	4.1 (2.2-7.0)	61 (1.3)	3.2 (2.5-4.1)
Pregnant or post partum	22 (1.8)	3.3 (2.1-5.0)	3 (0.6)	1.1 (0.2-3.1)	12 (6.1)	10.5 (5.4-18.4)	6 (2.3)	4.5 (1.7-9.9)
ACS								
Not pregnant or post partum	293 (0.9)	1.9 (1.7-2.1)	221 (1.2)	2.2 (1.9-2.5)	9 (1.7)	2.8 (1.3-5.4)	21 (0.5)	1.1 (0.7-1.7)
Pregnant or post partum	6 (0.5)	0.9 (0.3-2.0)	5 (1.0)	1.8 (0.6-4.1)	1 (0.5)	0.9 (0.0-4.9)	0	0.0 (0.0-2.8)
STSE-ACS^a^								
Not pregnant or post partum	107 (0.3)	0.7 (0.6-0.8)	71 (0.4)	0.7 (0.5-0.9)	5 (0.9)	1.6 (0.5-3.7)	11 (0.2)	0.6 (0.3-1.0)
Pregnant or post partum	3 (0.2)	0.5 (0.1-1.3)	2 (0.4)	0.7 (0.1-2.6)	1 (0.5)	0.9 (0.0-4.9)	0	0.0 (0.0-2.8)
Overall cardiovascular events								
Not pregnant or post partum	6694 (21.1)	43.6 (42.6-44.7)	3520 (18.4)	34.7 (33.5-35.8)	82 (15.0)	25.9 (20.6-32.1)	969 (21.1)	51.1 (47.9-54.4)
Pregnant or post partum	164 (13.6)	24.7 (21.1-28.8)	64 (13.0)	22.7 (17.5-29.0)	21 (10.7)	18.4 (11.4-28.1)	46 (17.7)	34.7 (25.4-46.3)
Death								
Not pregnant or post partum	3742 (11.8)	24.4 (23.6-25.2)	1305 (6.8)	12.9 (12.2-13.6)	23 (4.2)	7.3 (4.6-10.9)	1129 (24.6)	59.5 (56.1-63.1)
Pregnant or post partum	49 (4.1)	7.4 (5.5-9.8)	15 (3.0)	5.3 (3.0-8.8)	2 (1.0)	1.8 (0.2-6.3)	20 (7.7)	15.1 (9.2-23.3)

Abbreviations: CVT, cerebral venous thrombosis; ICH, intracerebral hemorrhage; PY, person-years; STSE-ACS, acute coronary syndrome with ST-segment elevation.

^a^ These events overlapped with overall acute coronary syndrome events.

Among the 76 women with recurrent stroke after a pregnancy-associated stroke, the distribution was 32 ISs (42.1%), 40 HSs (52.6%) (with 29 ICHs), and 4 CVTs (5.3%) (Table 1). The highest rates of stroke recurrence were observed when the first stroke was an ICH in women experiencing ICH during pregnancy or post partum (26.2; 95% CI, 24.0-28.7 per 1000 person-years) or a non–pregnancy-associated ICH (21.9; 95% CI, 14.7-31.4 per 1000 person-years) (Table 2). The highest rates of stroke recurrence were observed when the first stroke was an ICH in both women experiencing ICH during pregnancy or post partum (21.9; 95% CI, 14.7-31.4 per 1000 person-years) or a non–pregnancy-associated ICH (26.2; 95% CI, 24.0-28.7 per 1000 person-years) (Table 2). Finally, compared with the corresponding nonpregnant groups, pregnant women with a first CVT had a higher incident rate of venous thromboembolism (VTE) (10.5 [95% CI, 5.4-18.4] vs 4.1 [95% CI, 2.2-7.0]), and those with a first ICH had a higher incidence rate of both ICH (18.9 [95% CI, 12.2-27.9] vs 16.9 [95% CI, 15.1-18.8]) and VTE (4.5 [95% CI, 1.7-9.9] vs 3.2 [95% CI, 2.5-4.1]).

Recurrent stroke after pregnancy-associated stroke occurred in the first 30 days in 5 women (6.6%), between 30 days and 1 year of follow-up in 46 women (60.5%), and in 25 women (32.9%) after 1 year of follow-up. After non–pregnancy-associated stroke, recurrence occurred in 380 (13.9%) during the first 30 days, 1318 (48.1%) between 30 days and 1 year, and 1044 (38.1%) after 1 year of follow-up. Compared with pregnancy-associated stroke, the stroke recurrence rate was higher in non–pregnancy-associated stroke at the beginning of the follow-up, whereas the dynamic of stroke recurrence seemed similar after 1 year of follow-up (eFigure in Supplement 1), and women with pregnancy-associated stroke had a lower risk of recurrent stroke in multivariable analysis (HR, 0.63; 95% CI, 0.49-0.81) (Table 3). This result was driven by a lower risk of IS (HR, 0.53; 95% CI, 0.36-0.77) and subarachnoid hemorrhage (HR, 0.48; 95% CI, 0.27-0.85), without differences regarding other stroke causes. The HRs computed by the matched cohort analysis (eTable 3 in Supplement 1) were similar to those in the main analysis, although significance was lost for HRs related to subarachnoid hemorrhage.

**Table 3.** Hazard Ratios (95% CIs) of Stroke Recurrence, Cardiovascular Events, and Death During Follow-up in Women With a First Pregnancy-Associated Stroke vs Women With a First Non–Pregnancy-Associated Stroke According to the Type of First Stroke^a^

Outcome	Pregnancy-associated (n = 1204) and non–pregnancy-associated (n = 31 697) stroke (all types)		Pregnancy-associated (n = 482) and non–pregnancy-associated (n = 18 315) ischemic strokes			Pregnancy-associated (n = 242) and non–pregnancy-associated (n = 3792) intracerebral hemorrhage	
	Model 1^a^^,^^b^	Model 2^a^^,^^c^	Model 1^a^^,^^b^	Model 2^a^^,^^c^	Model 3^a^^,^^d^	Model 1^a^^,^^b^	Model 2^a^^,^^c^
All strokes	0.62 (0.48-0.78)	0.63 (0.49-0.81)	0.68 (0.47-1.00)	0.71 (0.47-1.05)	0.79 (0.50-1.26)	0.79 (0.53-1.17)	0.70 (0.45-1.08)
Ischemic stroke	0.46 (0.32-0.67)	0.53 (0.36-0.77)	0.65 (0.44-0.97)	0.70 (0.46-1.06)	0.80 (0.50-1.28)	0.64 (0.15-2.70)	0.80 (0.19-3.45)
Hemorrhagic stroke	0.81 (0.59-1.11)	0.73 (0.52-1.03)	1.04 (0.32-3.32)	0.78 (0.19-3.23)	0.68 (0.09-4.99)	0.80 (0.53-1.20)	0.68 (0.43-1.07)
Cerebral venous thrombosis	1.31 (0.30-5.73)	1.54 (0.34-6.95)	NA	NA	NA	NA	NA
Intracerebral hemorrhage	1.42 (0.96-2.09)	1.27 (0.83-1.95)	1.31 (0.31-5.50)	0.81 (0.11-5.99)	1.38 (0.18-10.40)	1.02 (0.66-1.56)	0.83 (0.51-1.34)
Subarachnoid hemorrhage	0.53 (0.31-0.90)	0.48 (0.27-0.85)	1.27 (0.17-9.71)	1.33 (0.17-10.30)	NA	0.77 (0.33-1.79)	0.59 (0.21-1.65)
Venous thromboembolism	1.85 (1.12-3.05)	2.02 (1.14-3.58)	0.53 (0.13-2.14)	0.74 (0.18-3.04)	0.59 (0.08-4.30)	1.71 (0.65-4.53)	1.48 (0.51-4.36)
Acute coronary syndrome	1.33 (0.57-3.07)	2.10 (0.89-4.94)	1.98 (0.79-4.95)	3.22 (1.26-8.19)	2.45 (0.74-8.08)	NA	NA
STSE-ACS^e^	2.10 (0.62-7.13)	3.93 (1.10-14.00)	2.43 (0.56-10.60)	4.99 (1.09-22.90)	5.74 (1.21-27.30)	NA	NA
Overall cardiovascular events	0.56 (0.48-0.65)	0.58 (0.49-0.69)	0.68 (0.53-0.87)	0.75 (0.57-0.97)	0.70 (0.50-0.96)	0.64 (0.47-0.87)	0.60 (0.43-0.84)
Death	0.43 (0.25-0.73)	0.42 (0.22-0.79)	0.79 (0.37-1.68)	0.59 (0.22-1.60)	0.89 (0.33-2.43)	0.30 (0.12-0.74)	0.30 (0.11-0.81)

Abbreviations: NA, not applicable; STSE-ACS, acute coronary syndrome with ST-segment elevation.

^a^ From Cox proportional hazards regression models using age as the time scale.

^b^ Model 1 unadjusted.

^c^ Model 2 excluded women with a history of ischemic heart disease or venous thromboembolism and was adjusted for history of hypertensive and antidiabetic medications, tobacco smoking, obesity, and Charlson Comorbidity Index score as a discrete covariate of 5 groups (0, 1, 2, 3, and >4).

^d^ Model 3 was similar to model 2 but excluded years before 2012 to adjust on revascularization therapy (intravenous thrombolysis, mechanical thrombectomy, or both vs none) during the first stroke.

^e^ These events overlapped with overall ACS events.

### Other Vascular Outcomes and Death

After pregnancy-associated stroke, 164 women (13.6%) experienced a hospitalization for a nonstroke vascular disease during the follow-up (incidence rate, 24.7; 95% CI, 21.1-28.8 per 1000 person-years). In addition, 49 women (4.1%) died (incidence rate, 7.4; 95% CI, 5.5-9.8 per 1000 person-years) (Table 2). The risks of cardiovascular events (HR, 0.58; 95% CI, 0.49-0.69) and death (HR, 0.42; 95% CI, 0.22-0.79) were lower in pregnancy-associated strokes than in non–pregnancy-associated strokes (Table 3). On the contrary, the risk of VTE was higher in pregnancy-associated strokes (HR, 2.02; 95% CI, 1.14-3.58) as was that of acute coronary syndrome with ST-segment elevation (HR, 3.93; 95% CI, 1.10-14.0). Of note, none of the acute coronary events occurred during the index pregnancy or post partum. The results of the matched cohort analysis (eTables 2 and 3 in Supplement 1) were consistent with the results of the primary analysis, although significance was lost for acute coronary syndrome with ST-segment elevation because of a large lack of statistical power.

### Vascular Outcomes and Death According to the Type of the Index Stroke

When considering the type of the index stroke, women with pregnancy-associated IS had a lower risk of other cerebrovascular events and cardiovascular events but a higher risk of acute coronary syndrome and ST-elevation myocardial infarction during follow-up than women with non–pregnancy-associated IS (Table 3). When restricting the analysis to IS as the index stroke, similar observations were found, even after adjustment on revascularization treatment, with the exception of the risk of death, which remained not significantly different between pregnancy-associated IS and non–pregnancy-associated IS. Women with pregnancy-associated ICH had a lower risk of all cardiovascular events and death compared with their nonpregnant counterparts.

### Stroke Recurrence, Cardiovascular Outcomes, and Death According to HDP

Hypertensive disorders of pregnancy occurred in 278 women with pregnancy-associated stroke (23.1%) (Table 1), 15 women with recurrent stroke (19.7%), 39 women with overall cardiovascular events (23.8%), and 10 women who died during follow-up (20.4%) (eTable 4 in Supplement 1). The complementary multivariate analysis that compared stroke recurrence between pregnancy-associated stroke with HDP and non–pregnancy-associated stroke showed similar HRs as the primary analysis (HR, 0.66 [95% CI, 0.51-0.86] vs HR, 0.63 [95% CI, 0.49-0.81]) (eTable 5 in Supplement 1) (ie, lower risk of stroke recurrence after pregnancy-associated stroke even with HDP). Higher risks of VTE and acute coronary syndrome with ST-segment elevation were still observed by comparing pregnancy-associated stroke with HDP (HR, 2.05 [95% CI, 1.14-3.70] and HR, 4.44 [95% CI, 1.24-15.90]) or without HDP (HR, 2.25 [95% CI, 1.19-4.25] and HR, 6.22 [95% CI, 1.72-22.60]) with non–pregnancy-associated stroke. Finally, no significance was reached for the risks of all the events of interest when comparing pregnancy-associated stroke with HDP and those without HDP (eTable 5 in Supplement 1).

### Stroke Recurrence During Subsequent Pregnancies or Post Partum

Among the 1204 women who had a stroke during pregnancy or post partum in France, only 194 (16.1%) had a subsequent pregnancy, with 72 (37.1%) having stroke after IS, 70 (36.1%) after HS, and 52 (26.8%) after CVT. Among them, 2 women (1.0%; 95% CI, 0.2%-4.1%) had another pregnancy-associated stroke. For these 2 women, stroke occurred in the postpartum period. One woman was aged 28 years at time of the first stroke, which was a CVT. She experienced an IS 2 weeks post partum after another pregnancy, which started only 3 months after the previous delivery. The other women had subarachnoid hemorrhage post partum as the index event at the age of 26 years and had an ICH post partum 4 years later during a subsequent pregnancy.

## Discussion

This cohort study found an incidence rate of recurrent stroke after a pregnancy-associated stroke of approximately 11 per 1000 person-years in France. Most recurrent events occurred in the first year after the pregnancy-associated stroke. The risk of subsequent ISs, subarachnoid hemorrhages, cardiovascular events, and death was lower in pregnancy-associated strokes compared with non–pregnancy-associated strokes, contrasting with a similar risk of ICH and CVT, and a higher risk of ST-elevation myocardial infarction and noncerebral VTE. Finally, after a pregnancy-associated stroke, the rate of stroke recurrence during a subsequent pregnancy was 1%.

In a previous study, no recurrence was noticed among women who had their first stroke during pregnancy.^4^ The number of patients enrolled was small (n = 28), and this study only considered IS, including CVT. Consistently, our results based on a larger population indicate that the risk of subsequent IS was low after pregnancy-associated stroke and even inferior to non–pregnancy-associated stroke in multivariable analyses. Several hypotheses could account for these findings. Pregnancy is associated with various physiologic changes, including hormonal modifications, hypercoagulability, and specific hypertensive disorders, which all favor IS occurrence. Consequently, many cases of pregnancy-associated IS can be regarded as situational of pregnancy and delivery and less related to patients’ risk factors that are traditionally associated with the risk of recurrence. In contrast, we observed a higher risk of acute coronary syndrome with ST-segment elevation in women who had pregnancy-associated stroke. Because pregnancy-associated strokes are partly linked to gestational hypertensive disorders, our finding could be consistent with previous studies that pointed out an excess of cardiovascular morbidity, especially ischemic heart disease, during follow-up of women with HDP.^11,12,13,14,15,16^ Nevertheless, we observed a lower risk of stroke recurrence in both women with pregnancy-associated stroke with HDP and those without compared with women with non–pregnancy-associated stroke. The inverse association between pregnancy and stroke recurrence as well as pregnancy and coronary risk remains unclear and needs further research. Similarly, we observed a lower risk of overall cardiovascular events and death in women with pregnancy-associated stroke compared with women with non–pregnancy-associated stroke despite higher risk of coronary heart disease and VTE. The protective effect of pregnancy on global stroke outcomes could be related to the better general health of pregnant women compared with nonpregnant women.^17^ In addition, the lower risk of death in women with pregnancy-associated stroke compared with non–pregnancy-associated stroke, particularly in the short term, could be related to the lower severity of pregnancy-associated strokes. This finding was previously observed in the American Heart Association’s Get With The Guidelines–Stroke Registry.^18,19^ These guidelines also highlight the need for careful coronary and venous monitoring of women with pregnancy-associated stroke.

In our study, the highest incidence rate of recurrence was observed for ICH in both pregnancy-associated and non–pregnancy-associated stroke, which is consistent with the literature highlighting higher stroke recurrence after ICH than other strokes.^20,21^ Of note, the recurrent cerebrovascular event after a first ICH was most often another ICH, thus indicating a common underlying cause of both episodes. Indeed, in this age group, the most frequent causes of ICH are cerebrovascular malformations, including cavernous angioma or arteriovenous malformation,^22^ which, when not treatable, carry an increased risk of recurrence.

Among 194 women who had a subsequent pregnancy, we reported a second pregnancy-associated stroke in 2 cases. The type of the index stroke was CVT and subarachnoid hemorrhage in these women. One woman had CVT during the index pregnancy and IS during the subsequent pregnancy. The ISCVT (International Study on Cerebral Vein and Dural Sinus Thrombosis)–2 PREGNANCY Study^23^ of 119 women who had CVT reported 1 recurrent CVT and 2 noncerebral VTEs during 82 pregnancies among 47 women during a median follow-up of 169 months.

A recent systematic review included 5 studies (but not the ISCVT) of pregnancy-associated strokes and aimed to assess recurrence rates of stroke during subsequent pregnancies.^6^ This review reported a higher rate of subsequent pregnancy after pregnancy-related stroke (50%) than in our study (16.1% [194 of 1204]). The authors highlighted this relatively high proportion based on a limited number of published cases. The recurrence rate of stroke was estimated at 2% (1 woman of the 55 included). This stroke was a first-trimester CVT in a woman with a history of CVT during pregnancy who had sickle cell disease as an underlying condition.

### Strengths and Limitations

The major strength of our study was the exhaustive inclusion of pregnancy-associated stroke in France for a large period, the enrollment of nonpregnant counterparts, and investigation of associated recurrent events. This approach allowed us to observe recurrent events even during subsequent pregnancies.

This study also has several limitations. One limitation was that the retrospective design was linked to the use of an administrative database from which it is not possible to have detailed information on specific variables of interest other than those predefined in the database. Nevertheless, no information bias exists with administrative recording. We aimed to include a population of women with incident stroke. However, we were not able to assess stroke history before 2006 because no reliable information was provided in the hospital database we used. Other limitations resided in the restricted medical information on stroke in our databases. For instance, we had no information regarding stroke severity and corresponding evaluation score, such as the National Institutes of Health Stroke Scale or Rankin Scale. Moreover, health administrative databases do not precisely record IS mechanisms. Therefore, we were not able to clarify underlying pathology in pregnancy- and non–pregnancy-associated strokes. In addition, analyses by type of stroke had a lack of statistical power because of the small number of women with pregnancy-associated stroke with a documented subtype of stroke. This lack of statistical power also affected some outcomes measured (acute coronary syndrome), thus limiting the interpretation of the results. Subsequent hospitalization for stroke in the first 30 days after the index hospitalization for stroke was considered as an early stroke recurrence, but we cannot exclude that this was a subsequent hospitalization linked to the index stroke. However, only a small proportion of subsequent hospitalizations for stroke occurred before 30 days. We also cannot exclude a residual confounding effect linked to the lack of recording of other risk factors and cardiovascular medications after stroke. In addition, despite a median follow-up of 5.6 years in pregnancy-associated strokes and 4.7 years in non–pregnancy-associated strokes, this time frame may be insufficient for observing more subsequent pregnancies, leading to an underestimation of recurrent stroke during subsequent pregnancies. These follow-up periods might also not be enough to evaluate the risk of future cardiovascular diseases.

## Conclusion

In this cohort study, the recurrence of ISs, overall long-term cardiovascular outcomes, and death after pregnancy-associated stroke remained lower than in women with non–pregnancy-associated stroke except for VTE and myocardial infarction. These results suggest that pregnancy-associated risk factors for stroke can disappear after pregnancy and the postpartum period, reducing the risk of recurrence thereafter. The higher risk of VTE and acute myocardial infarction after pregnancy-associated stroke compared with non–pregnancy-associated stroke highlights the additional risk associated with pregnancy regarding these events.

## Notes

Supplement 1. **eMethods.** Supplemental Methods **eTable 1.** Characteristics of Women Experiencing Pregnancy-Associated Strokes and Non-Pregnancy-Associated Stroke According to the Type of First Stroke **eTable 2.** Characteristics of Women Experiencing Pregnancy-Associated Strokes (N = 1,186) and Matched Non-Pregnancy-Associated Stroke (N = 5,930) **eTable 3.** Hazard Ratios With 95% Confidence Intervals (HR [95% CI]) of Stroke Recurrence, Cardiovascular Events and Death During the Follow-up, in Women With a First Pregnancy-Associated Stroke vs Matched Women With a First Non-Pregnancy-Associated Stroke (1:5) **eTable 4.** Hypertensive Disorders of Pregnancy (HDP) Prevalence (%) Among Women With Pregnancy-Associated Stroke According to the Occurrence of Events of Interest During the Follow-up **eTable 5.** Hazard Ratios With 95% Confidence Intervals (HR [95% CI]) of Stroke Recurrence, Cardiovascular Events and Death During the Follow-up in Women With a First Pregnancy-Associated Stroke According to the Hypertensive Disorders of Pregnancy (HDP) and Compared to Women With a First Non-Pregnancy-Associated Stroke **eFigure.** Kaplan-Meier Curves for Stroke Recurrence, Cardiovascular Events and Survival in Women With a Pregnancy-Associated Stroke and Non-Pregnancy-Associated Stroke

Supplement 2.
Data Sharing Statement
