# Supplementary material for: Comparison of Stroke Recurrence, Cardiovascular Events, and Death Among Patients With Pregnancy-Associated vs Non–Pregnancy-Associated Stroke
Source: JAMA Netw Open. 2023 Jun 7;6(6):e2315235. doi: 10.1001/jamanetworkopen.2023.15235 (PMC10248736; doi:10.1001/jamanetworkopen.2023.15235)
Supplement: Supplement 1. — eMethods. Supplemental Methods eTable 1. Characteristics of Women Experiencing Pregnancy-Associated Strokes and Non-Pregnancy-Associated Stroke According to the Type of First Stroke eTable 2. Characteristics of Women Experiencing Pregnancy-Associated Strokes (N = 1,186) and Matched Non-Pregnancy-Associated Stroke (N = 5,930) eTable 3. Hazard Ratios With 95% Confidence Intervals (HR [95% CI]) of Stroke Recurrence, Cardiovascular Events and Death During the Follow-up, in Women With a First Pregnancy-Associated Stroke vs Matched Women With a First Non-Pregnancy-Associated Stroke (1:5) eTable 4. Hypertensive Disorders of Pregnancy (HDP) Prevalence (%) Among Women With Pregnancy-Associated Stroke According to the Occurrence of Events of Interest During the Follow-up eTable 5. Hazard Ratios With 95% Confidence Intervals (HR [95% CI]) of Stroke Recurrence, Cardiovascular Events and Death During the Follow-up in Women With a First Pregnancy-Associated Stroke According to the Hypertensive Disorders of Pregnancy (HDP) and Compared to Women With a First Non-Pregnancy-Associated Stroke eFigure. Kaplan-Meier Curves for Stroke Recurrence, Cardiovascular Events and Survival in Women With a Pregnancy-Associated Stroke and Non-Pregnancy-Associated Stroke [file jamanetwopen-e2315235-s001.pdf]

Supplementary Online Content

Béjot Y, Olié V, Lailler G, et al. Comparison of stroke recurrence, cardiovascular events, and death among patients with pregnancy-associated vs non–pregnancy-associated stroke. *JAMA Netw Open*. 2023;6(6):e2315235. doi:10.1001/jamanetworkopen.2023.15235

eMethods. Supplemental Methods

eTable 1. Characteristics of Women Experiencing Pregnancy-Associated Strokes and Non-Pregnancy-Associated Stroke According to the Type of First Stroke

eTable 2. Characteristics of Women Experiencing Pregnancy-Associated Strokes (N=1,186) and Matched Non-Pregnancy-Associated Stroke (N=5,930)

eTable 3. Hazard Ratios[a] With 95% Confidence Intervals (HR [95% CI]) of Stroke Recurrence, Cardiovascular Events and Death During the Follow-up, in Women With a First Pregnancy-Associated Stroke vs. Matched Women With a First Non-Pregnancy-Associated Stroke (1:5)

eTable 4. Hypertensive Disorders of Pregnancy (HDP) Prevalence (%) Among Women With Pregnancy-Associated Stroke According to the Occurrence of Events of Interest During the Follow-up

eTable 5. Hazard Ratios[a] With 95% Confidence Intervals (HR [95% CI]) of Stroke Recurrence, Cardiovascular Events and Death During the Follow-up in Women With a First Pregnancy-Associated Stroke According to the Hypertensive Disorders of Pregnancy (HDP) and Compared to Women With a First Non-Pregnancy-Associated Stroke

eFigure. Kaplan-Meier Curves for Stroke Recurrence, Cardiovascular Events and Survival in Women With a Pregnancy-Associated Stroke and Non-Pregnancy-Associated Stroke

This supplementary material has been provided by the authors to give readers additional information about their work.

© 2023 Béjot Y et al. *JAMA Network Open.*

eMethods. Supplemental Methods

Stroke was defined by the following International Classification of Diseases-Tenth Revision codes (ICD-10) that were registered as the main diagnosis of the entire hospital stay or one of the medical units where the patient stayed: hemorrhagic stroke, including intracerebral hemorrhage (ICH) (I61), subarachnoid hemorrhage (SAH) (I60) and other non-traumatic intracranial hemorrhage (I62); ischemic stroke (IS) (I63-I64; excluding I63.6); and CVT (I63.6, O22.5 and O87.3).

Follow-up and Outcomes

Cardiovascular events gathered any hospitalization for a disease of the chapter IX of the ICD-10 called "Diseases of the Circulatory System". Besides overall cardiovascular events, we also focused on venous thromboembolism (VTE) (including both pulmonary embolism (ICD-10 codes I26, O88.2) and deep venous thrombosis (ICD-10 codes I80-I82 (except I80.0), O22.3, O87.1), and acute coronary syndrome (ICD-10 codes I20.0, I21-I24), including hospitalization for acute coronary syndrome with ST segment elevation (STSE-ACS) (ICD-10 code I21.1-I21.3).

Women's characteristics

The following characteristics were used: age, sex, and the social deprivation index of the town of living[1,2]. This deprivation index was calculated for each French town based on the median household income, the percentage high school graduates in the population aged 15 years and older, the percentage blue-collar workers in the active population, and the unemployment rate. Each woman included in the study were assigned to the social deprivation index of their city of residence. A history of cardiovascular disease was searched in the past 5 years according to the hospital diagnosis of cardiovascular disease or long-term disease status using ICD-10 codes. A history of cardiovascular drug treatment in the past 12 months was recorded for each woman before the index stroke regarding the following medications (identified using "Anatomical Therapeutic Chemical Classification" [ATC]): antihypertensive, lipid-lowering agents, antidiabetics, heparin treatments, anticoagulants, antiplatelet agents and antiarrhythmic. Charlson comorbidity index was calculated as a sum of weighted major comorbidities for each women hospitalized for stroke[3,4]. For pregnancy-associated stroke, some specific information were recorded: gestational diabetes and hypertensive disorders of pregnancy (HDP) which included gestational hypertension, pre-eclampsia/eclampsia, and HELLP Syndrome.

Statistical Analyses

Three models were built for the multivariate analyses of stroke recurrence, cardiovascular incidence and death occurrence in women with pregnancy-associated stroke and non-pregnancy-associated stroke. Model 1 was the unadjusted one. For Model 2, women with a history of ischemic heart disease and VTE were excluded in order to capture incident ischemic heart disease and VTE, and the model was adjusted for a history of antihypertensive medication treatment, antidiabetics, tobacco smoking, obesity and Charlson Index score as a discrete covariate of five groups (0,1,2,3, and >4). A third model was computed for IS only: this model was restricted to population study after 2012 and adjusted for acute revascularization treatment (IV thrombolysis or mechanical thrombectomy (MT)) (Model 3). For this last model, the limitation to the population study enrolled after 2012 with IS was constrained by the unavailability of information on IV thrombolysis before 2012. MT or bridging therapy were also recorded.

To assess the potential role of HDP in stroke recurrence, cardiovascular incidence and death occurrence, three complementary analyses were performed and presented in supplemental material. In the first complementary analysis, we have compared the risk of the event of interest between pregnancy-associated stroke with HDP only and non-pregnancy-associated stroke. In the second complementary analysis, we have compared the risks of the events of interest between pregnancy-associated stroke without HDP and non-pregnancy-associated stroke. In the third complementary analysis, the risks of events of interest were compared between pregnancy-associated stroke with HDP and pregnancy-associated stroke without HDP.

eReferences

1. Rey G, Jougla E, Fouillet A, Hémon D. Ecological association between a deprivation index and mortality in France over the period 1997 - 2001: variations with spatial scale, degree of urbanicity, age, gender and cause of death. BMC Public Health. 2009;9:33.

2. Tuppin P, Rudant J, Constantinou P, et al. Value of a national administrative database to guide public decisions: From the systeme national d'information interregimes de l'Assurance Maladie (SNIIRAM) to the systeme national des donnees de sante (SNDS) in France. Revue d'epidemiologie et de sante publique. 2017;65 Suppl 4:S149-s167.
3. Banna A, Chaignot C, Blotière PO, Weill A, Ricordeau P, Alla F. Score de Charlson à partir des données du Sniiram chaînées au PMSI : faisabilité et valeur pronostique sur la mortalité à un an. Revue d'epidemiologie et de sante publique. 2013;61:S9.
4. Quan H, Li B, Couris CM, et al. Updating and validating the Charlson comorbidity index and score for risk adjustment in hospital discharge abstracts using data from 6 countries. American journal of epidemiology. 2011;173(6):676-682.

© 2023 Béjot Y et al. *JAMA Network Open.*

eTable 1: Characteristics of women experiencing pregnancy-associated strokes and non-pregnancy-associated stroke according to the type of first stroke

	Type of first stroke								
	Ischemic stroke			Cerebral venous thrombosis			Intracerebral hemorrhage		
	Pregnancy status at the 1st stroke			Pregnancy status at the 1st stroke			Pregnancy status at the 1st stroke		
	Pregnant/post-partum (n=493)	Non-pregnant (n=19,162)	p	Pregnant/post-partum (n=196)	Non-pregnant (n=545)	p	Pregnant/post-partum (n=260)	Non-pregnant (n=4,592)	p
Mean age/median, years	31.8/32	39.8/42	**<.001**	29.0/29	33.3/34	**<.001**	31.9/32	39.0/42	**<.001**
Age groups, %			**<.001**			**<.001**			**<.001**
15-29 years	174 (35.3)	2,438 (12.7)		111 (56.6)	209 (38.4)		89 (34.2)	778 (16.9)	
30-39 years	276 (56.0)	4,977 (26.0)		76 (38.8)	160 (29.3)		148 (56.9)	1,120 (24.4)	
40-49 years	43 (8.7)	11,747 (61.3)		9 (4.6)	176 (32.3)		23 (8.9)	2,694 (58.7)	
Tobacco smoking, %	79 (16.0)	4,187 (21.9)	**0.002**	11 (5.6)	49 (9.0)	0.14	15 (5.8)	450 (9.8)	**0.03**
Obesity, %	38 (7.7)	1,921 (10.0)	0.09	9 (4.6)	43 (7.9)	0.12	16 (6.2)	296 (6.5)	0.85
In-hospital death	3 (0.6)	376 (2.0)	**<.001**	1 (0.5)	7 (1.3)	**<.001**	15 (5.8)	700 (15.2)	**<.001**
Stroke symptoms, %									
Plegia (hemiplegia, paraplegia)	190 (38.5)	7,425 (38.8)	0.93	20 (10.2)	88 (16.2)	0.04	92 (35.4)	1,575 (34.3)	0.72
Aphasia	141 (28.6)	5,370 (28.0)	0.78	13 (6.6)	84 (15.4)	**0.002**	61 (23.5)	914 (19.9)	0.16
Epilepsy	22 (4.5)	538 (2.8)	**0.03**	30 (15.3)	109 (20.0)	0.15	35 (13.5)	436 (9.5)	**0.04**
Migraine	32 (6.5)	1,090 (5.7)	0.45	6 (3.1)	22 (4.0)	0.54	3 (1.2)	89 (1.9)	0.37
Other migraine symptoms	16 (3.3)	243 (1.3)	**<.001**	10 (5.1)	49 (9.0)	0.08	16 (6.2)	88 (1.9)	**<.001**
Headache	56 (11.4)	1,604 (8.4)	**0.02**	42 (21.4)	143 (26.2)	0.18	63 (24.2)	811 (17.7)	**0.007**
History of cardiovascular treatment in the year prior to index stroke, %									
Antihypertensives	26 (5.4)	3,186 (17.8)	**0.002**	6 (3.1)	24 (4.7)	0.35	10 (4.0)	713 (17.3)	**<.001**
Lipid-lowering	5 (1.0)	1,044 (5.8)	**<.001**	0 (0.0)	8 (1.6)	0.08	1 (0.4)	176 (4.3)	**0.003**
Antidiabetics	11 (2.3)	848 (4.7)	**<.001**	6 (3.1)	8 (1.6)	0.19	1 (0.4)	122 (3.0)	**0.02**
Oral anticoagulants	9 (1.9)	404 (2.3)	**0.01**	1 (0.5)	21 (4.1)	**0.01**	3 (1.2)	101 (2.4)	0.212
All anticoagulants	20 (4.2)	563 (3.2)	0.22	14 (7.3)	25 (4.9)	0.22	5 (2.0)	152 (3.7)	0.172
Antiplatelets	13 (2.7)	864 (4.8)	**0.03**	2 (1.0)	5 (1.0)	0.94	1 (0.4)	112 (2.7)	**0.022**
Antiarrhythmics	5 (1.0)	236 (1.3)	0.59	0 (0.0)	2 (0.4)	0.38	0 (0.0)	90 (2.2)	**0.02**
Comorbidities (5 yearts of history), %									
Charlson index score (number of comorbidities), %			0.09			**<.001**			**0.008**
0	301 (61.1)	10,798 (56.4)		175 (89.3)	428 (78.5)		161 (61.9)	2,564 (55.8)	
1	2 (0.4)	514 (2.7)		2 (1.0)	9 (1.7)		4 (1.5)	124 (2.7)	

© 2023 Béjot Y et al. *JAMA Network Open.*

2	167 (33.9)	6,682 (34.9)		18 (9.2)	92 (16.9)		87 (33.5)	1,487 (32.4)	
3	7 (1.4)	445 (2.3)		1 (0.5)	6 (1.1)		1 (0.4)	97 (2.1)	
4+	16 (3.3)	723 (3.8)		0 (0.0)	10 (1.8)		7 (2.7)	320 (7.0)	
Ischemic heart disease	10 (2.0)	904 (4.7)	0.005	0 (0.0)	2 (0.4)	0.40	1 (0.4)	90 (2.0)	0.07
Acute coronary syndrome	6 (1.2)	377 (2.0)	0.23	0 (0.0)	2 (0.4)	0.40	1 (0.4)	63 (1.4)	0.17
Atrial fibrillation	11 (2.2)	510 (2.7)	0.56	0 (0.0)	4 (0.7)	0.89	1 (0.4)	58 (1.3)	0.21
All heart rhythm disorders	25 (5.1)	1,234 (6.4)	0.22	4 (2.0)	12 (2.2)	0.30	7 (2.7)	265 (5.8)	0.04
Heart failure	7 (1.4)	535 (2.8)	0.07	0 (0.0)	3 (0.5)	0.16	3 (1.2)	129 (2.8)	0.11
Pulmonary embolism	12 (2.4)	363 (1.9)	0.39	4 (2.0)	23 (4.2)	0.62	4 (1.5)	76 (1.7)	0.89
All venous thromboembolism diseases	27 (5.5)	772 (4.0)	0.11	20 (10.2)	49 (9.00)	0.80	13 (5.0)	214 (4.7)	0.80
All hospitalization for circulation diseases (ICD-10 code I)	36 (7.3)	3,346 (17.5)	<.001	21 (10.7)	62 (11.4)	0.008	23 (8.8)	828 (18.0)	<.001
Pregnancy-related disorders, %									
Hypertensive disorders of pregnancy	76 (15.4)	-	-	22 (11.2)	-	-	76 (29.2)	-	-
Gestational hypertension	47 (9.5)	-	-	12 (6.1)	-	-	47 (18.1)	-	-
Pre-eclampsia/Eclampsia	44 (8.9)	-	-	14 (7.1)	-	-	48 (18.5)	-	-
HELLP syndrome	7 (1.4)	-	-	3 (1.5)	-	-	18 (6.9)	-	-
Gestational diabetes	43 (8.7)	-	-	23 (11.7)	-	-	20 (7.7)	-	-

Abbreviations: ICD-10, international classification of disease-10th revision

© 2023 Béjot Y et al. *JAMA Network Open.*

eTable 2: Characteristics of women experiencing pregnancy-associated strokes (n=1,186) and matched non-pregnancy-associated stroke (n=5,930)

	No.(%)					
	All women with a first stroke			Women with recurrent stroke		
	Pregnancy status at the 1st stroke			Pregnancy status at the 1st stroke		
	Pregnant/post-partum (N=1,186)	Non-pregnant (N=5,930)	p-value	Pregnant/post-partum (N=74)	Non-pregnant (N=475)	p-value
Mean age/median, years	31.6/32	31.6/32	1.00	32.8/34	31.7/32	0.13
Age groups, %						
15-29 years	442 (37.3)	2,210 (37.3)		21 (28.4)	176 (37.1)	
30-39 years	640 (54.0)	3,200 (54.0)		45 (60.8)	254 (53.5)	
40-49 years	104 (8.8)	520 (8.8)		8 (10.8)	45 (9.5)	
Quintile of social deprivation of the town of living, %			0.95			0.43
Quintile 1 (the least deprived)	204 (18.8)	1,058 (19.2)		17 (25.4)	97 (22.5)	
Quintile 2	235 (21.5)	1,155 (21.0)		15 (22.4)	80 (18.6)	
Quintile 3	210 (19.4)	1,023 (18.6)		14 (20.9)	68 (15.8)	
Quintile 4	206 (18.7)	1,075 (19.5)		9 (13.4)	93 (21.6)	
Quintile 5 (the most deprived)	233 (21.5)	1,190 (21.6)		12 (17.9)	93 (21.6)	
Missing	*98*	*429*		*7*	*44*	
Subtype of the first stroke, %						
Ischemic stroke, excluding CVT	500 (42.2)	3,658 (61.7)	**<.001**	30 (40.5)	262 (55.2)	**0.02**
CVT	208 (17.5)	175 (3.0)	**<.001**	4 (5.4)	8 (1.7)	**0.04**
Hemorrhagic stroke (All)	480 (41.8)	2,159 (36.4)	**0.007**	41 (55.4)	208 (43.8)	0.06
ICH	261 (22.0)	879 (14.8)	**<.001**	29 (39.2)	94 (19.8)	**<.001**
Subarachnoid hemorrhage	240 (20.2)	1,265 (21.3)	0.26	15 (20.3)	124 (26.1)	0.28
In-hospital death	28 (2.4)	223 (3.4)	0.25	-	-	-
	Stroke symptoms, %					
Plegia (hemiplegia, paraplegia)	314 (26.5)	1,511 (25.5)	0.47	34 (45.0)	153 (32.2)	**0.02**
Aphasia	218 (18.4)	1,149 (19.4)	0.43	22 (29.7)	108 (22.7)	0.19
Epilepsy	109 (9.2)	279 (4.7)	**<.001**	9 (12.2)	27 (5.7)	**0.04**
Migraine	47 (4.0)	341 (5,8)	**0.01**	6 (8.1)	18 (3.8)	0.09
Other migraine symptoms	56 (4.7)	132 (2.2)	**<.001**	4 (5.4)	16 (3.4)	0.38
Headache	222 (18.7)	988 (16.7)	0.09	14 (18.9)	78 (16.4)	0.59
	History of cardiovascular treatment in the year prior to index stroke, %					
Antihypertensives	50 (4.3)	377 (6.8)	**0.001**	4 (5.7)	39 (8.8)	0.40

© 2023 Béjot Y et al. *JAMA Network Open.*

Lipid-lowering	7 (0.6)	69 (1.3)	0.06	0 (0.0)	6 (1.4)	0.33
Antidiabetics	22 (1.9)	70 (1.3)	0.09	1 (1.3)	7 (1.6)	0.93
Oral anticoagulants	14 (1.2)	80 (1.5)	0.53	0 (0.0)	9 (2.0)	0.23
All anticoagulants	48 (4.2)	119 (2.2)	**<.001**	0 (0.0)	11 (2.5)	0.18
Antiplatelets	19 (1.6)	107 (1.9)	0.50	2 (2.9)	13 (2.9)	0.97
Antiarrhythmics	5 (0.4)	40 (0.7)	0.27	1 (1.4)	5 (1.1)	0.83
Heparin	43 (3.7)	42 (0.8)	**<.001**	0 (0.0)	4 (0.9)	0.43
	Comorbidities (5 years of history), %					
Charlson index score (number of weighted comorbidities), %						
0	853 (71.9)	4,265 (71.9)	1.00	39 (52.7)	308 (64.8)	0.06
1	9 (0.8)	45 (0.8)		0(0.0)	6 (1.3)	
2	301 (25.4)	1,505 (25.4)		33 (44.6)	146 (30.7)	
3	8 (0.7)	40 (0.7)		1 (1.3)	8 (1.7)	
4+	15 (1.3)	75 (1.3)		1(1.3)	7 (1.5)	
Ischemic heart disease	12 (1.0)	127 (2.1)	**0.01**	1 (1.3)	12 (2.5)	0.54
Acute coronary syndrome	7 (0.6)	42 (0.7)	0.65	1 (1.3)	4 (0.8)	0.67
Atrial fibrillation	14 (1.2)	66 (1.1)	0.84	0 (0.0)	11 (2.3)	0.19
All heart rhythm disorders	42 (3.5)	247 (4.2)	0.32	2 (2.7)	25 (5.3)	0.34
Heart failure	15 (1.3)	91 (1.5)	0.48	1 (1.3)	8 (1.7)	0.83
Pulmonary embolism	20 (1.7)	69 (1.2)	0.14	1 (1.3)	6 (1.3)	0.95
All venous thromboembolism diseases	69 (5.8)	183 (3.1)	**<.001**	3 (4.1)	19 (4.0)	0.98
All hospitalization for circulation diseases (ICD-10 code I)	98 (8.3)	674 (11.4)	**0.002**	5 (6.8)	82 (17.3)	**0.02**

Abbreviations: CVT, cerebral venous thrombosis; ICH, intracerebral hemorrhage; ICD-10, international classification of disease-10th revision; in bold: p-value <0.05.

© 2023 Béjot Y et al. *JAMA Network Open.*

eTable 3: Hazard ratios[a] with 95% confidence intervals (HR [95% CI]) of stroke recurrence, cardiovascular events and death during the follow-up, in women with a first pregnancy-associated stroke vs. matched women with a first non-pregnancy-associated stroke (1:5)

Outcomes	HR[a] between pregnancy-associated (n=1,186) and matched non-pregnancy-associated (n=5,930) women at time of stroke (**all types**)	
	HR[a] from model 1[b]	HR[a] from model 2[c]
All strokes	**0.75[0.57-0.99]**	**0.75 [0.57-0.99]**
Ischemic stroke	**0.56[0.37-0.84]**	**0.59 [0.38-0.91]**
Hemorrhagic stroke	1.00[0.70-1.44]	0.97 [0.67-1.42]
Cerebral venous thrombosis	2.01[0.37-11.1]	3.31 [0.45-24.2]
Intracerebral hemorrhage	**1.62[1.02-2.57]**	1.51 [0.93-2.46]
Subarachnoid hemorrhage	0.68[0.37-1.26]	0.70 [0.37-1.30]
Venous thromboembolism	**2.07[1.12-3.84]**	1.98 [0.96-4.11]
Acute coronary syndrome	2.48[0.84-7.31]	3.23 [0.82-12.7]
STSE-ACS[d]	3.81[0.60-24.1]	5.35 [0.44-65.4]
All cardiovascular events	**0.73[0.61-0.87]**	**0.74 [0.62-0.90]**
Death	0.65[0.35-1.21]	0.51 [0.25-1.04]

Abbreviations: HR, Hazard Ratio; STSE-ACS, Acute coronary syndrome with ST segment elevation; bold: p-value<0.05.

[a]from Cox proportional hazard models using age as time-scale.

[b]Model 1 unadjusted;

[c]Model 2 excluded women with a history of ischemic heart disease or venous thrombo-embolism and was adjusted on history of hypertensive and antidiabetic medications, tobacco smoking, obesity and Charlson Index score as a discrete covariate of five groups (0,1,2,3 and >4).

[d]these events overlapped with overall acute coronary syndrome events.

© 2023 Béjot Y et al. *JAMA Network Open.*

eTable 4: Hypertensive disorders of pregnancy (HDP) prevalence (%) **among women with pregnancy-associated stroke** according to the occurrence of events of interest during the follow-up

Events during the follow-up	Events observed (yes/no (N))		All HDP	Gestational Hypertension	Pre-eclampsia/Eclampsia	HELLP Syndrome
All strokes	Yes (n=76)	N	15	9	7	2
		%	19.7	11.8	9.2	2.6
	No (n=1,128)	N	263	181	142	38
		%	23.3	16.1	12.6	3.4
	p-value		*0.474*	*0.331*	*0.387*	*0.729*
Ischemic strokes	Yes (n=35)	N	8	6	3	1
		%	22.9	17.1	8.6	2.9
	No (n=1,169)	N	270	184	146	39
		%	23.1	15.7	12.5	3.3
	p-value		*0.974*	*0.823*	*0.488*	*0.876*
Hemorrhagic strokes	Yes (n=42)	N	7	3	4	1
		%	16.7	7.1	9.5	2.4
	No (n=1,162)	N	271	187	145	39
		%	23.3	16.1	12.5	3.4
	p-value		*0.315*	*0.118*	*0.568*	*0.729*
Cerebral venous thrombosis	Yes (n=2)	N	0	0	0	0
		%	0.0	0.0	0.0	0.0
	No (n=1,202)	N	278	190	149	40
		%	23.1	15.8	12.4	3.3
	p-value		*0.438*	*0.540*	*0.595*	*0.793*
Intracerebral hemorrhage	Yes (n=30)	N	4	1	3	1
		%	13.3	3.3	10.0	3.3
	No (n=1,174)	N	274	189	146	39
		%	23.3	16.1	12.4	3.3
	p-value		*0.199*	*0.058*	*0.689*	*0.997*
Subarachnoïd hemorrhage	Yes (n=14)	N	3	1	2	0
		%	21.4	7.1	14.3	0.0
	No (n=1,190)	N	275	189	147	40
		%	23.1	15.9	12.4	3.4
	p-value		*0.882*	*0.373*	*0.827*	*0.486*
Venous thromboembolism disease	Yes (n=22)	N	4	3	2	0
		%	18.2	13.6	9.1	0.0
	No (n=1,182)	N	274	187	147	40
		%	23.2	15.8	12.4	3.4
	p-value		*0.582*	*0.781*	*0.637*	*0.380*
Acute coronary syndrome	Yes (n=6)	N	1	0	1	0
		%	16.7	0.0	16.7	0.0
	No (n=1,198)	N	277	190	148	40
		%	23.1	15.9	12.4	3.3
	p-value		*0.708*	*0.288*	*0.749*	*0.649*
STSE-ACS	Yes (n=3)	N	0	0	0	0
		%	0.0	0.0	0.0	0.0

© 2023 Béjot Y et al. *JAMA Network Open.*

	No (n=1,201)	N	278	190	149	40
		%	23.2	15.8	12.4	3.3
	p-value		*0.342*	*0.453*	*0.515*	*0.748*
Overall cardiovascular events	Yes (n=164)	N	39	27	19	5
		%	23.8	16.5	11.6	3.1
	No (n=1,040)	N	239	163	130	35
		%	23.0	15.7	12.5	3.4
	p-value		*0.821*	*0.797*	*0.741*	*0.834*
Death	Yes (n=49)	N	10	2	10	5
		%	20.4	4.1	20.4	10.2
	No (n=1,155)	N	268	188	139	35
		%	23.2	16.3	12.0	3.0
	p-value		*0.649*	*0.022*	*0.081*	*0.006*

Abbreviations: HDP, Hypertensive Disorders of Pregnancy; STSE-ACS, Acute coronary syndrome with ST segment elevation; bold: p-value<0.05.

© 2023 Béjot Y et al. *JAMA Network Open.*

eTable 5: Hazard ratios[a] with 95% confidence intervals (HR [95% CI]) of stroke recurrence, cardiovascular events and death during the follow-up in women with a first pregnancy-associated stroke according to the hypertensive disorders of pregnancy (HDP) and compared to women with a first non-pregnancy-associated stroke

	HR[a] between pregnancy-associated stroke with HDP (n=278) and non-pregnancy-associated stroke (n=31,697) (all types of first stroke)		HR[a] between pregnancy-associated stroke without HDP (n=926) and non-pregnancy-associated stroke (n=31,697) (all types of first stroke)		HR[a] between pregnancy-associated stroke with HDP (n=926) and without HDP (n=926) (all types of first strokes)	
Outcomes	HR[a] from model 1[b]	HR[a] from model 2[c]	HR[a] from model 1[b]	HR[a] from model 2[c]	HR[a] from model 1[b]	HR[a] from model 2[c]
All strokes	**0.60[0.47-0.78]**	**0.66[0.51-0.86]**	**0.63[0.47-0.84]**	**0.69[0.52-0.92]**	0.81[0.44-1.52]	0.83[0.44-1.55]
Ischemic stroke	**0.49[0.33-0.72]**	**0.58[0.39-0.85]**	**0.45[0.29-0.72]**	**0.53[0.34-0.84]**	1.32[0.56-3.07]	1.37[0.58-3.20]
Hemorrhagic stroke	0.73[0.52-1.03]	0.75[0.53-1.07]	0.82[0.57-1.19]	0.85[0.59-1.24]	0.51[0.20-1.32]	0.52[0.20-1.35]
Cerebral venous thrombosis	1.61[0.36-7.25]	1.67[0.37-7.55]	2.05[0.45-9.28]	2.10[0.46-9.53]	-	-
Intracerebral hemorrhage	1.24[0.81-1.90]	1.33[0.86-2.05]	1.43[0.91-2.24]	1.50[0.94-2.38]	0.43[0.13-1.44]	0.43[0.13-1.46]
Subarachnoid hemorrhage	**0.48[0.27-0.86]**	**0.48[0.26-0.87]**	**0.47[0.24-0.92]**	**0.50[0.26-0.98]**	1.04[0.28-3.86]	1.08[0.29-4.05]
Venous thromboembolism	**1.91[1.08-3.39]**	**2.05[1.14-3.70]**	**1.97[1.04-3.72]**	**2.25[1.19-4.25]**	0.92[0.25-3.34]	0.91[0.25-3.33]
Acute coronary syndrome	1.86[0.79-4.36]	**2.35[1.00-5.54]**	2.12[0.84-5.39]	**2.63[1.03-6.71]**	0.50[0.06-4.26]	0.51[0.06-4.52]
STSE-ACS[d]	3.30[0.93-11.7]	**4.44[1.24-15.9]**	**4.73[1.32-17.0]**	**6.22[1.72-22.6]**	-	-
All cardiovascular events	**0.56[0.47-0.66]**	**0.61[0.51-0.72]**	**0.56[0.46-0.67]**	**0.62[0.51-0.75]**	1.05[0.71-1.54]	1.08[0.73-1.59]
Death	**0.37[0.19-0.68]**	**0.41[0.21-0.80]**	**0.34[0.16-0.73]**	**0.43[0.21-0.92]**	1.46[0.37-5.69]	1.52[0.38-6.07]

Abbreviations: HR, Hazard Ratio; STSE-ACS, Acute coronary syndrome with ST segment elevation; bold: p-value<0.05.

[a]from Cox proportional hazard models using age as time-scale.

[b]Model 1 unadjusted;

[c]Model 2 excluded women with a history of ischemic heart disease or venous thrombo-embolism and was adjusted on history of hypertensive and antidiabetic medications, tobacco smoking, obesity and Charlson Index score as a discrete covariate of five groups (0,1,2,3 and >4)

[d]these events overlapped with overall acute coronary syndrome events.

© 2023 Béjot Y et al. *JAMA Network Open.*

eFigure . Kaplan-Meier Curves for stroke recurrence, cardiovascular events and survival in women with a pregnancy-associated stroke and non-pregnancy-associated stroke.

A) Stroke recurrence

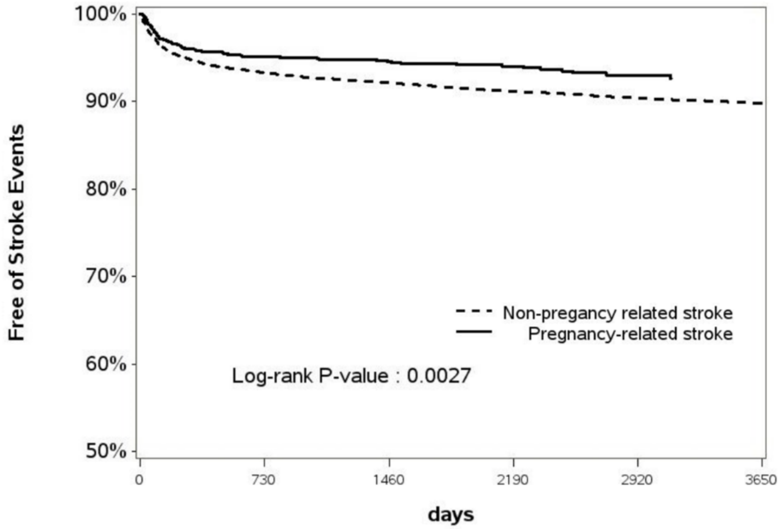

No. at risk

Non-pregnancy group	24,097	17,938	12,442	7,074	2,264
Pregnancy group	1,037	783	563	328	89

B) Overall cardiovascular events

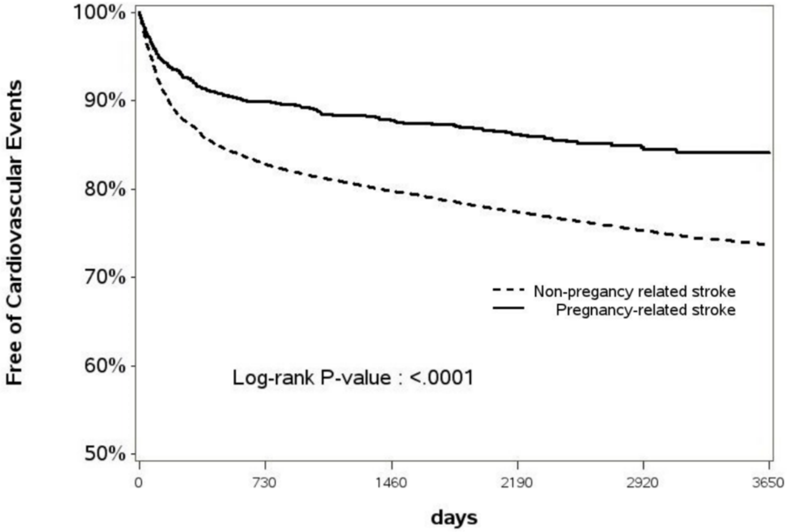

C) Survival

© 2023 Béjot Y et al. *JAMA Network Open.*

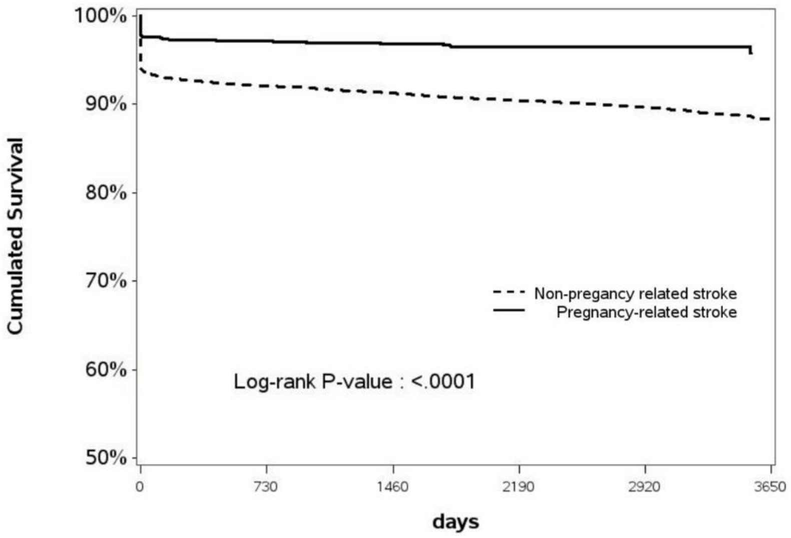

© 2023 Béjot Y et al. *JAMA Network Open.*
