# Supplementary material for: Comparison of Stroke Recurrence, Cardiovascular Events, and Death Among Patients With Pregnancy-Associated vs Non–Pregnancy-Associated Stroke
Source: JAMA Netw Open. 2023 Jun 7;6(6):e2315235. doi: 10.1001/jamanetworkopen.2023.15235 (PMC10248736; doi:10.1001/jamanetworkopen.2023.15235)
Supplement: Supplement 2. — Data Sharing Statement [file jamanetwopen-e2315235-s002.pdf]

Data Sharing Statement

Béjot. Comparison of Stroke Recurrence, Cardiovascular Events, and Death Among Patients With Pregnancy-Associated vs Non–Pregnancy-Associated Stroke. *JAMA Netw Open*. Published June 07, 2023. doi:10.1001/jamanetworkopen.2023.15235

Data
Data available: No

Additional Information
Explanation for why data not available: Data will not be shared. According to the French governmental regulations and the National Ethics Committee, no patient consent was required. The databases used in the study contained pseudonymised patient information. Furthermore, full access to the SNDS which includes the French National Hospital Databases is granted to the National Agency for Public Health (Santé Publique France) by decree (regulatory decision DE-2011-078).
